# Should Deceased Donation be Morally Preferred in Uterine Transplantation Trials?

**DOI:** 10.1111/bioe.12247

**Published:** 2016-02-01

**Authors:** Nicola Williams

**Keywords:** uterine transplants, quality of life transplants, autonomy, beneficence, non‐maleficence, transplantation ethics, reproductive ethics

## Abstract

In recent years much research has been undertaken regarding the feasibility of the human uterine transplant (UTx) as a treatment for absolute uterine factor infertility (AUFI). Should it reach clinical application this procedure would allow such individuals what is often a much‐desired opportunity to become not only social mothers (via adoption or traditional surrogacy arrangements), or genetic and social mothers (through gestational surrogacy) but mothers in a social, genetic and gestational sense. Like many experimental transplantation procedures such as face, hand, corneal and larynx transplants, UTx as a therapeutic option falls firmly into the camp of the quality of life (QOL) transplant, undertaken with the aim, not to save a life, but to enrich one. However, unlike most of these novel procedures – where one would be unlikely to find a willing living donor or an ethics committee that would sanction such a donation – the organs to be transplanted in UTx are potentially available from both living and deceased donors.

In this article, in the light of the recent nine‐case research trial in Sweden which used uteri obtained from living donors, and the assertions on the part of a number of other research teams currently preparing trials that they will only be using deceased donors, I explore the question of whether, in the case of UTx, there exist compelling moral reasons to prefer the use of deceased donors despite the benefits that may be associated with the use of organs obtained from the living.

## Introduction

Late 2014 marked the end of a long and hard‐fought race between teams of researchers across the world to perform the first ‘successful’ experimental human uterine transplant (UTx), a procedure designed as a treatment option for women with absolute Uterine Factor Infertility (UFI). This condition affects approximately one in every 500 women worldwide of childbearing age^1^ and is an umbrella term covering infertility problems in individuals who either lack a uterus as a result of congenital abnormality or a previous hysterectomy, or possess a uterus but due to physiological or anatomical abnormalities are unable to conceive or sustain gestation.^2^ Such women *cannot* be assisted by more mainstream assisted reproductive technologies such as IVF as these are designed to treat difficulties in conception, not gestation. Thus, a successful treatment for UFI has been called the ‘last frontier’^3^ in reproductive medicine, allowing women with UFI the opportunity to become, not only social mothers as they already may via adoption or traditional surrogacy arrangements, or social and genetic mothers, as they can be through gestational surrogacy arrangements, but mothers in a social, genetic, *and* gestational sense.

To date only three notable ‘sets’ of attempts at human UTx have been performed. The first took place in Saudi Arabia in 2000,^4^ the second in Turkey in 2011,^5^ and the third in Sweden where nine procedures were undertaken between September 2012 and April 2013.^6^ A number of other teams including those based in Japan,^7^ France,^8^ the UK,^9^ Australia^10^ and the US,^11^ are also preparing to embark on their first trials of this procedure and the UK team received ethics approval for a 10‐case trial in September of this year.^12^ Whilst no live births resulted from the first two attempts, although pregnancy was achieved briefly in the Turkish case,^13^ four live births have now been recorded in Sweden and more are expected to follow in late 2015 and early 2016.^14^

The live birth of a child after human UTx has provided hope and the possibility of additional choice for many women with UFI who wish to become mothers but who are unwilling, unable, or may prefer not to resort to adoption or surrogacy, for various reasons – personal, pragmatic, moral and legal. However, although UTx *may* offer assistance to those unable to gestate their own offspring, it also raises a number of interesting ethical questions. Some of these relate to the practice itself,^15^ others to its current status as an experimental procedure,^16^ and others still to more generic concerns regarding the ethics of assisted reproductive technologies. Although such lines of enquiry warrant close attention, the scope of this article is more limited and an answer to a more specific question is sought. This question is as follows: *Taking as a basis the view that UTx is both a valuable prospective treatment option for UFI and there exist no compelling ethical arguments telling against its performance generally: is the use of deceased donors morally preferable in UTx research?*

There are two main reasons why this question has been chosen. The first is that there exists considerable disagreement among researchers as to whether living or deceased donors should be preferred. The second is the fact that of existing QOL transplantation procedures UTx is the only procedure, barring that of the ovary transplant, for which the use of living donors has been proposed, sanctioned by an ethics committee and utilized. As such, with an answer to the above question in mind, the first part of this article examines the views expressed by researchers performing or preparing to perform UTx regarding their preference for deceased or living donors while the second looks to the possible moral reasons that may tell against a preference for living donors.

## Living vs. Deceased Donation in UTX Trials ‐ The Perspectives of the Research Teams

Of the 11 notable human UTx procedures performed so far, all but one has utilized living donors. The first, in Saudi Arabia, used a living, unrelated donor already undergoing a radical hysterectomy due to the presence of benign ovarian cysts.^17^ The second, in Turkey, used a deceased (brain dead) multi‐organ donor^18^ and the final nine, in Sweden, used only live donors, seven of whom were closely genetically related (mothers/sisters) to the recipient.^19^ Yet, despite the recent successes of the Swedish team there has been much debate amongst those currently preparing trials regarding the question of whether living or deceased donation should be preferred. Thus, whilst some teams are considering emulating the Swedish model such as teams based in Spain^20^ and Japan^21^ others such as in France,^22^ the UK,^23^ Belgium^24^ and at least one of the four US teams based in New York^25^ have stated that their preference lies with the use of deceased donors.

So, why is this the case? Why are the research teams currently unable to agree on such a foundational matter? Within this section, the reasons provided by researchers on both sides of the debate are outlined and examined.

### A preference for living donors

Those who prefer living donors appeal to two distinct kinds of benefits as informing their choice. They suggest, firstly, that the use of living donors will increase the likelihood of the transplant's success, and secondly, that living donors should be preferred, at least at this stage in research, for pragmatic reasons.

In terms of increased likelihood of success, members of both the Swedish and Japanese teams have noted that, just as living related donation in kidney and partial liver transplantation leads to better patient outcomes and long‐term graft survival rates, and decreases the need for strong immunosuppressive regimes, it is highly likely that the same benefits will be obtained from living‐related donation in UTx.^26^ In cases of unrelated donation they also suggest living donors should be preferred on the basis of results of long‐term studies of graft survival rates after kidney donation showing that kidneys obtained from living unrelated donors still provide better patient outcomes than those from deceased donors. Brannstrom et al. suggest this may be because brain death induces systemic inflammation that negatively affects organ quality^27^ and Kisu et al. propose that, as removal of non‐vital organs must occur after the removal of vital organs for transplantation, inevitable increases in warm ischemia time may reduce organ quality and function.^28^ The Swedish team also suggest that the use of living donors lessens the risk of transplanting uteri that are unsuitable for gestation, allowing ‘for meticulous diagnostic work‐up of the uterine graft to exclude pathologies that could interfere with fertility potential’ and ‘the choice of a uterine graft that has proved its functionality in terms of normal pregnancies’.^29^

The Swedish team also provide three pragmatic reasons for their preference. In regards to the first, they suggest that there are too few deceased donors to make UTx using deceased donors an option in Sweden.^30^ Statistics regarding posthumous donation in Sweden are amongst the lowest in Europe, even after a switch to an opt‐out policy in 1996. Thus, whilst it is unlikely that there would be too few deceased donors for a small‐scale research trial in Sweden, should UTx reach clinical application it is likely the case that demand for deceased donor uteri would outstrip supply. Secondly, they suggested that due to their experience of living donation in animal studies, the use of living donors would be preferable for them, although this might not be the case elsewhere.^31^ Finally, they suggested that dependent on research context there may be additional practical reasons to prefer the use of living donors. Brannstrom notes:

…our team with surgeons from three continents had to plan the exact dates of our surgeries well in advance. Furthermore, our hospital board required that all surgeries be done during weekends in operating theatres that were usually closed on weekends. Deceased donor UTx would not be an option under these conditions.^32^

### A preference for deceased donors – Turkey, The US and the UK

Those who prefer the use of deceased donors also offer reasons for their preference, which, in certain cases, contradict those provided by teams preferring living donation. As with those the Swedish and Japanese teams provide, these have primarily been based in considerations of pragmatism and the suggestion that the use of deceased donor uteri is just as, if not *more likely*, to prove successful.

Regarding the likelihood of success, despite the only successful pregnancies in humans occurring in recipients of uteri obtained from living donors, some who prefer the use of deceased donors have pointed to the fact that during the animal model stage of UTx research, and in the Saudi case, a major barrier to successful transplantation has been necrosis caused by thrombosis in the vessels supplying blood to the uterus. It was found that in order to reduce the likelihood of thrombosis longer lengths of vasculature should be used; these are most easily and effectively obtained from deceased donors as the large abdominal vena cava can be removed from deceased but not living donors.^33^ Some view this consideration as decisive and Smith and Del Priori – the leaders of the UK and New York teams ‐ have stated categorically that: ‘the uterus for transplantation is best obtained from a deceased donor and not a live donor.’^34^ Others draw attention to the fact that, despite the claims of the Swedish and Japanese teams that living donation should be preferred in terms of long‐term graft survival, such benefits are questionable as the procedure is designed to be ephemeral (removed after one or two pregnancies). The Turkish team, for example have looked at the results of a meta‐analysis of data regarding graft survival rates in organ and composite tissue transplants which shows that, whilst organs obtained from the living do tend to survive longer than those from deceased donors due to tissue matching and organ quality factors, these show only *very* minimal effects on five‐year graft survival rates^35^ and thus provide little reason to prefer living donors.

A number of practical reasons have also been offered in favour of the use of deceased donors, focusing mainly on there being a larger potential pool of deceased donors for UTx, the surgery for retrieval of organs being far less complex and there being no risk of harm to the donor. Indeed, although the latter may be couched as an ethical reason to prefer deceased over living donation of uteri (a reason discussed in forthcoming sections) there is also a practical element to this consideration. Removal of the risk of serious complications and mortality in donors by using only uteri obtained from the deceased negates the risk of research being forcibly abandoned due to this. Del Priore and Gudipudi note:

…each time a living donor suffers a severe complication from donation, even of a life saving liver transplant, this inevitable occurrence typically shuts down even the best transplantation programs and predicts that as such the death of a living uterus donor would end this nascent era of human uterus transplantation.^36^

## Should Deceased Donation be Ethically Preferred in UTX Research?

As might thus be garnered, whether deceased or living donors should be preferred on grounds of pragmatism or increased likelihood of success in UTx trials is difficult to answer with certainty. Yet, despite this, some may hold that *even if* the use of living donors can be shown to be more practicable/successful, deceased donors would still be preferable on the basis of ethical considerations such as the harms that may be suffered by living donors, concerns regarding donor consent, and the possibility of regret. Such considerations will now be explored and examined.

### Harms to uterus donors

Discussions in the UTx literature regarding whether living or deceased donors should be preferred *all* have one thing in common: an acknowledgement of the fact, and an expression of concern that, whilst retrieval of uteri from the deceased poses no risk of donor harm, this is not the case with living donation. Retrieval from the living *necessitates* physical harm to the donor and includes small but not insignificant risks of long‐term morbidity and mortality thought similar to, or only slightly higher than that of a total abdominal hysterectomy.^37^ These include possible damage to the ureter and intestines,^38^ complications associated with general anaesthesia, psychological problems such as loss of gender identity, and a risk of sexual dysfunction associated with hysterectomy such as a decrease in sexual satisfaction, interest, arousal and orgasm.^39^

For some, notably the Japanese and Swedish teams, whilst the use of living donors adds ‘another key element into a risk‐benefit analysis concerning uterus transplantation,’^40^ the possible harms that may befall living donors are thought justified, *provided* certain background conditions are met. Yet for others, such as the International Federation of Gynaecology and Obstetrics (FIGO), that the retrieval of a uterus from a living donor ‘necessitates a relatively major surgery with its own risk of complications’^41^ constitutes reason enough to deem the procedure ‘ethically inappropriate’.^42^

Appeals to harm, however, are not likely to be as straightforward as the prescriptions of FIGO might suggest. For, although the retrieval of uteri from living donors causes donor harm and provides no medical benefit, holding an obligation of non‐maleficence as absolute seems far too strong and is not in keeping with current medical practice. The living donation of non‐vital organs and parts of certain vital organs has been permitted in certain circumstances since the dawn of the transplantation era and the use of living donors for single kidney transplantation is now so commonplace that in the UK over one third of kidneys for transplantation are so obtained.^43^ Phase one clinical research trials of medicine and certain other interventions also routinely use healthy volunteers – that is, individuals with no current need for treatment with the drug/intervention concerned – in order to test for safety and possible side effects before subsequent phases in specific clinical populations. In such trials volunteers expose themselves to small risks of sometimes significant harm and the dominant position among those concerned with the ethics of clinical trials involving healthy subjects is that, provided certain background conditions are met, exposure to such risks is justified.

Thus, unless the above practices are to be roundly condemned, mere acknowledgement of the harms and risks associated with the living donation of uteri cannot, for reasons of consistency, prove a decisive reason against this practice. Instead, it must be shown that *UTx trials are somehow different from accepted instances of living donation and clinical trials using human volunteers*: that one or some number of the grounds often invoked as justifying their use in these contexts (and, indeed, many others), fails to apply in the case of UTx.

#### Justifying harm in the contexts of living vital organ donation and clinical research using healthy volunteers

A number of criteria have been proposed as providing justification, when met, for the imposition of harm in cases of living donation and participation in research trials. Of these, appeals to one or some number of the following three tend to dominate:

- ***Valid Consent*:** The vast majority of scholars concerned with research/transplantation ethics hold that a necessary condition for justified living donation/research participation is the provision of valid consent on the part of donors/participants.^44^ For some libertarian scholars this requirement is also thought to prove sufficient as it is held that – provided an individual is in possession of the capacities and information required for autonomous choice *and* gives his ‘free, voluntary, and undeceived consent’^45^ – he may just as easily consent to causing himself/being caused harm as he may to furnishing himself with benefits.^46^ For most however, valid consent, whilst necessary, is not sufficient for justified donation/participation and is thus combined with one or both of the conditions (b) and (c) that follow.
- ***Favourable harm‐benefit ratio (aggregative)*:** Another common proposal is that, necessary for justified research participation/donation involving living volunteers, is that their use can be expected to provide an all‐things‐considered benefit for *all* those with a stake in the research/donation.^47^ This view tends to have its basis in the consequentialist view that the infliction of harm on one/some will be justifiable where it can reasonably be expected to secure greater benefits for others.^48^ Whilst on the simplest version of this view all that is required for justified donation/participation is the *production of a net benefit,* 49 for most, if not all, proponents of this view: that a net benefit results is not sufficient to justify the harms inflicted. Thus, (b) also tends to require that: the harms inflicted are:
  - *Necessary* to secure the benefit sought^50^ (which involves both careful consideration of possible *and similarly beneficial alternatives* to the use of living donors/volunteers such as the use of deceased donors or other treatment options in transplantation and other research models in clinical research);
  - *Minimized* only to those necessary.^51^
    As in (a), those who hold both the weaker and stronger versions of this view may variously suggest that it is both necessary *and* sufficient for justified research/donation or that, whilst individually necessary, (b) is not sufficient and must be paired with (a) and/or (c).
- ***Sufficiently good harm‐benefit ratio (non‐aggregative: donor/volunteer focused)*:** Whilst rarely suggested as a necessary and sufficient condition for justified living donation and research, held in tandem with (a) and/or (b) is the view that key to justified donation/participation lies in the *production of a sufficiently good ratio of harm‐benefit for the donor him/herself*. What counts as favourable on this view differs greatly dependent on who one asks, but normally comes in the following forms. Within transplantation ethics literature some suggest that that living donation is only justifiable where donors *receive a net benefit or, at least, are not harmed on balance* by their donation.^52^ Others suggest, less strongly, that a threshold should be placed on the *extent* to which a donor/volunteer may be harmed as a result of their donation, suggesting that although donation procedures and experiments which result in net harm to the donor may be permitted there is a point at which the harms become unacceptably grave.^53^ Although none have specified this threshold, it is generally suggested that the most risky of currently performed living donation procedures (living liver lobe, partial lung transplants) fall somewhere *near* the limits of acceptable harms and risks and that procedures such as living second kidney^54^ and heart donation,^55^ fall outside of them. Less strongly still, and more commonly, especially within the research ethics literature, is the suggestion that although a favourable or neutral balance of benefit to burden is unnecessary for justified living donation/research,^56^ attempts should be made, where possible, to maximize the benefits donors and volunteers receive as a result of their donation/participation.^57^

#### Are the harms to living donors in UTx justifiable?

With the above in mind, it seems that if the living donation of uteri is to be deemed ‘ethically inappropriate’ on the grounds of the harms and risks imposed on donors, such a complaint is most likely to take one or some number of the following forms:

- ***Consent*:** The harms associated with living uterus donation are unjustifiable as they are not *of a kind or intensity* to which the donor may provide valid consent.
- ***Favourable Harm – Benefit Ratio (donor/recipient)*:** In the case of UTx the potential benefits to the recipient and future recipients fail to outweigh the harms the donor may suffer and/or are unnecessary in order to achieve the desired benefit.
- ***Balance of Harms and Benefit (donor)*:** In the case of UTx the harms to the donor are too grave, and the benefits: not maximised and/or fail to compensate for the harms she may suffer.

Of the complaints listed above, (a) – suggesting that the harms associated with uterus donation may not be of a kind or intensity to which a donor may provide valid consent – is undoubtedly the weakest. For, although there is much debate within the literature on the ethics of living donation regarding the limits of both respect for autonomy and of autonomy itself – and such debates provide valuable insight when considering the question of whether individuals should be permitted to donate second kidneys or hearts – engagement with this debate is highly unlikely to provide sufficient reason to prefer deceased over living donation in UTx. The living uterus donor does not, after all, offer to subject herself to anything like the risks associated with such procedures and does not even offer subjection to the kinds of risks associated with already accepted forms of living donation such as single kidney transplants. Consequently, although the risks of living donation in UTx may well have been underplayed by the research teams performing/preparing UTx trials, it seems improbable that consideration of the limits of autonomy will provide good moral reason to prefer the use of deceased donors in the case of UTx.

Similarly, *for those who hold there is more involved in justified living organ donation or participation in medical research than a donor's/participant's informed and voluntary consent,* 58 an appeal to (c) is also unlikely to provide reason to condemn the use of living donors in UTx. Although overall benefit for donors cannot be guaranteed, as above there is little reason to presume that the harms she will suffer as a result of her donation will fall below some permissible threshold, or to suggest that living uterus donors *cannot* receive an all‐things‐considered benefit from their donation. Donor benefit is often viewed as sufficient to allow non‐directed living kidney donation by strangers and such donation provides only the benefits associated with selfless acts of helping such as increased self‐esteem.^59^ Thus, given that infertility and an inability to gestate may have a devastating impact on individual welfare, with those experiencing it often feeling distress, depression, loss of gender identity and an enduring sense of incompleteness and grief,^60^ it seems there is good reason to assume that the benefits accrued to living donors, especially where donors and recipients share a close personal relationship, will prove sufficient. Such benefits may include but are not limited to: knowing that one has done all one can to provide a loved one the opportunity to fulfil a strong and deeply held desire and to alleviate the suffering brought on by the frustration of that desire, as well as fulfilling one's own desire to become a grandmother/aunt and watch said loved one experience pregnancy and birth etc.^61^ Indeed, even in cases of altruistic living donation by strangers it is easy to imagine a woman who appreciates the value of gestation and has already completed her own family gaining *significant* pleasure and pride from knowledge that she had assisted another in fulfilling her desire to gestate and grow her family.^62^

If donor benefit may outweigh donor harm in the case of UTx, it may be safely assumed that so too will recipient/possible future recipient benefit *often* straightforwardly outweigh donor harm. After all, the benefits received by such parties, where the transplant proves successful, are likely to be far greater in both intensity and duration than those accrued to the donor. Similarly, should UTx not prove a viable treatment option for UFI, the knowledge gained from the trial may provide some comfort to those experiencing it, helping them to accept their inability to gestate, and choose among options already available to them as all possible treatment avenues will have been exhausted.

Whether living donation in UTx satisfies the requirements of necessity and harm minimization may, however, be questioned. With reference to supply and demand, for example, it does not seem to be the case that there is *currently* a need *–* as in other living donation contexts – to resort to the use of living donors in UTx. Demand for donor uteri has, after all, reached only 11 uteri at this point and transplantations can be performed at a relatively leisurely pace given that UTx is both an experimental procedure and aims to treat a condition that, although distressing, is not life limiting.^63^ Of course, this is not to say that should UTx reach clinical application there may not be a shortage of deceased donor uteri for transplantation. Thus, it may be suggested that *because* shortage in clinical practice has been foreseen there are good reasons to use both the living and deceased donor concepts in research.

Appeals to the second practical sense of necessity – the provision of additional benefits – may also be used to justify living donation in this case. Due to the experimental nature of the procedure, such benefits are currently uncertain and it may be found in the future that even *if* living donation in UTx is more likely to prove successful or leads to better patient outcomes, the additional benefits that the use of living donors provides may not be sufficient to outweigh the harms of donation in the face of *slightly less beneficial* but *far less risky* alternatives. However, given that there are good scientific reasons backing up the claim that the use of organs obtained from living donors may prove more efficacious, research into this possibility, using both living related/unrelated and deceased donors does seem justifiable provided researchers use minimally invasive methods of retrieval, by for example, exploring laparoscopic and robot assisted donations in living volunteers.

### Consent, voluntariness and regret

As noted previously, consideration of the limits of respect for individual autonomy will not provide a solid reason to prefer the use of deceased donors. Living donors may just as easily provide consent to uterus retrieval as they may to the retrieval of organs such as kidneys and liver lobes, provided they possess both the capacities and information required to make an informed choice.

Despite this, it may be suggested that it is still preferable to use deceased donors in UTx in order to *avoid risking* the possibility that living donors in UTx may be used in instances where – due to poor institutional procedures/safeguards, and/or despite appearances to contrary – their consent to donate is not fully informed and/or wholly voluntary. After all, living donation in UTx does risk the possibility that donors may be coerced or manipulated into donation by those holding stakes in their donation and that they may also feel an internal pressure to donate even in the absence of actively coercive or manipulative acts.

That living donation in UTx poses such risks is acknowledged in the literature by parties on both sides of the debate. Kisu et al. note that, unlike deceased donors, ‘living donors…have the burden that they may experience pressure to give and take the uterus.’^64^ Lefkowitz et al. suggest similarly: ‘If the donor is a live donor, issues arise with informed consent of the donor relating to organ donation in general and specific to the donation of a uterus.’^65^ Caplan holds too that the use of living donors increases the risk of coercion and adds: ‘There could also be potential problems if a family member initially consents to being a uterus donor and then changes her mind.’^66^ Catsanos et al. echo such sentiments but suggest not all forms of living uterus donation are equally risky, holding that altruistic donation by strangers and those already undergoing a hysterectomy can reduce concerns over coerced consent’,^67^ although without the immunological benefits associated with living related donation.

Even where valid and informed consent is provided, concerns have also surfaced regarding the possibility of donor regret. This has mainly been articulated by blog commentators on UTx but is also acknowledged by Catsanos et al. who note:

There is… a risk for uterus donors in that a uterus is only expendable if the potential donor is unequivocally certain that she will not now nor in the future desire another pregnancy herself… some of the women who responded to news of UTx had chosen hysterectomy as the solution to a medical problem, thinking they had completed their families, only to find themselves in a new relationship and desirous of having children with their new partner.^68^

For some, the possibility of regret might prove less relevant in determining the acceptability of living transplants. Regret is often an inevitable part of life and paternalistic attempts to save individuals from future harms by curtailing their freedom in the present often prove self defeating, as individuals are often in the best, albeit non‐ideal, position to know how likely they are to regret their choices.

However, regardless of whether one views regret as relevant to determinations of the ethical permissibility of the use of living donors in UTx, it should be noted that concerns regarding coercion and regret are not unique to living uterus donation. They arise in multiple contexts ranging from the exciting, such as the living donation of organs and tissues generally and surrogacy arrangements, to the mundane, such as marriages, employment, loans, house purchases. In such contexts however, the dominant position of those concerned with their ethics is *not* the implementation of a precautionary approach to risk prevention *but instead* the provision of robust mechanisms and safeguards designed to protect against and thus minimize the occurrence of instances where the consent of a donor has been undercut and donor regret. Certainty regarding consent of another can *never* be obtained given that it is both a propositional attitude^69^ and it is impossible to fully don the mental mantle of another. However, steps can be taken to significantly reduce the risk of coercion, manipulation and internal compulsion and regret.

With this in mind it is suggested that researchers in UTx trials draw on pre‐existing criteria for informed consent from both transplantation and research contexts when designing living UTx trials and consider implementing additional safeguards relevant to UTx. Such requirements are thus likely to include but may not be limited to:

1. The provision of verbal and written information from research teams and demonstration of understanding on the part of the donor regarding:
  - The medical, social and financial risks of donation;
  - The likelihood of success and the risk of morbidity and mortality for the recipient;
  - Possible alternative sources of donor organs for the recipient such as deceased and living donors already undergoing hysterectomies *and* alternative means of begetting children such as adoption and surrogacy.
2. Repeated and consistent attestation on the part of the donor regarding a desire to donate.
3. Confidential evaluation of donor by physicians and psychologists separate to the research trial and absent the presence of family members, friends and members of the research team with a stake in the donation to ensure competence, voluntariness and donor advocacy. This may include allowing and supporting the telling of a ‘lie’ regarding incompatibility or medical contraindications from the beginning of assessment until the transplant procedure in order to remove fear of blame from family members if they do not wish to donate but are worried about the consequences of failing to do so.
4. The use only of donors who are older, post‐menopausal and/or already undergoing a hysterectomy in order to minimize the potential for donor regret.^70^

In cases where centres lack the resources or institutional stability^71^ required to afford such protections to donors, the possibility of a failure to obtain informed consent and regret may provide adequate reason to suggest that only deceased donors should be used. Indeed, this may also be the case dependent on the location of the trial in question. In lower income and more pro‐natalistic societies Mumtaz and Levay note that risks of coercion and manipulation of potential uterus donors may be greater than in other societies due to a combination of less well‐developed/funded mechanisms for informed and voluntary consent and a higher value afforded to parenthood. They note, for example, that in Pakistan childlessness is often viewed as socially unacceptable and that women who cannot produce children are liable to experience physical and emotional abuse from their husbands and in‐laws. As such, women who have already completed their families or who are single and well past ‘marriageable’ age may experience far greater pressure both externally and internally to donate to relatives/friends who find themselves in this precarious position.^72^

Although consideration of the above may lead some to hold that it may always be morally preferable to use deceased donors in order to *bypass* concerns of coercion, manipulation and internal compulsion, it has been suggested that the use of deceased donors in UTx trials may, in certain circumstances, raise similar problems. Caplan, for example, raises an interesting point regarding the appropriateness of *presuming consent* to donate a uterus in those who have signed a donor card prior to their death. He asks:

…is this really enough? Few, if any, American women ever thought that the uterus might be one of the organs considered for donation when they signed a donor card. A woman might not prove as willing to donate her uterus as she would be to donate her heart or liver.^73^

Caplan's question highlights the importance of taking into account *both* the novelty and relatively unknown status of UTx and the fact that individuals often hold complex preferences regarding donation such that they may be less willing to donate non‐vital rather than vital organs after their death and may be more averse still to donating reproductive organs. That individuals who have joined the organ donor register may hold complex preferences regarding donation is illustrated easily by referring to the UK NHS Transplant Activity Report. Although the report shows 88% of registered donors are willing to donate all organs and tissues posthumously, a significant minority of 12% are selective regarding the types of organs and tissues they will donate. Of this minority, 88%, for example, are unwilling to donate their corneas, and 23% refuse to donate pancreases and hearts.^74^

Despite this, the stakes involved in ensuring the consent of living donors do seem greater than those involved in deceased donation. Unless we hold that our identities are grounded in some separately existing, noumenal entity – such as a soul or Cartesian ego – the suggestion that a person may be caused harm by acts done to their bodies after death is nonsensical. After all, ‘since for the time when [they] are, death is not present; and for the time when death is present, [they] are not.’^75^ Of course, this is not to say there are no moral reasons to respect an individual's wish not to donate their organs posthumously. The knowledge, after all, that a preference against post‐humous organ donation is liable to be ignored may cause that individual great distress and so too would such removal distress family members posthumously should they know and/or share his preference. Yet, that we are concerned with ensuring consent on the part of the deceased to donate seems, if anything, to lend strength to the claim that – where consent from the living to donate is in question – the use of deceased donors *should still* be preferred. For, the negative consequences arising from failure to obtain informed consent in cases of living donation are of significantly more gravity.

## Conclusion

Within this article I have sought to provide an answer to the question of whether deceased donation should be preferred in UTx trials given that the use of living donors in UTx trials is both necessarily harmful to donors *and* risks the possibility of failures to attain fully informed consent and donor regret. In general, my response to this question is positive: there exist *pro tanto* reasons to prefer the use of deceased donors.

However, whilst this is so, as with all *pro tanto reasons,* a moral preference for deceased donation only goes so far. The imposition of harm on living volunteers/donors in research and organ transplantation contexts *may be* – and indeed, often is – justified by appeals to the value of respect for individual autonomy, a favourable balance of harm and benefit between donors and recipients/stakeholders, and a favourable balance of harm and benefit for donors themselves. Thus, unless we are to roundly condemn existing organ donation and research practices, there seems little reason to suggest that, *at this stage in research,* the harms and risks imposed on living donors in UTx trials cannot be justified by appeals to such considerations. Similarly, as concerns regarding consent and regret plague virtually all research and transplantation contexts, and few suggest a precautionary approach should be implemented in order to protect against such risks, there seems little reason to assume precaution should prevail in the case of UTx, provided robust mechanisms and safeguards are implemented that reduce such risks.

Of course, this is not to say that, once trials are completed, the use of living donors will still be justifiable. For, should it be the case that there is both no shortage of deceased donor uteri *and* the use of living donors is no more likely/only slightly more likely to prove successful, those who hold that justified living donation requires a favourable balance of harm‐benefit for donors may claim, with good reason, that living donors should not be utilized.

## Footnotes

1

L. Johannesson et al. The Future of Human Uterus Transplantation. Women's Health 2014; 10: 455. 10.2217/whe.14.2225259905

2

A. Lefkowitz , M. Edwards & J. Balayla . The Montreal Criteria for the Ethical Feasibility of Uterine Transplantation. Transpl Int 2012; 25: 439. 2235616910.1111/j.1432-2277.2012.01438.x

3

M. Brannstrom et al. Uterus Transplantation: Animal Research and Human Possibilities. Fertil Steril 2012; 97: 1269. 2254299010.1016/j.fertnstert.2012.04.001

4

W. Fageeh et al. Case Report ‐ Transplantation of the Human Uterus. Int J Gynaecol Obstet 2002; 76: 245–251. 1188012710.1016/s0020-7292(01)00597-5

5

M.E. Akar et al. Clinical Pregnancy after Uterus Transplantation. Fertil Steril 2013; 100: 1358–1363. 2383011010.1016/j.fertnstert.2013.06.027

6

M. Brannstrom et al. The First Clinical Uterus Transplantation Trial: A Six‐Month Report. Fertil Steril 2014; 101: 1228–1236. 2458252210.1016/j.fertnstert.2014.02.024

7

I. Kisu et al. Current Status of Uterus Transplantation in Primates and Issues for Clinical Application. Fertil Steril 2013;100: 287–288. 10.1016/j.fertnstert.2013.03.00423557761

8
L. Smith. Woman with Womb Transplant Gives Birth to Healthy Baby in Medical Landmark. *The Independent*, 4 October 2014. Available at: http://www.independent.co.uk/life-style/health-and-families/health-news/woman-with-womb-transplant-gives-birth-to-healthy-baby-in-medical-landmark-9774267.html [accessed 29 June 2015].

9
B. Czub. Pregnancy Hope for Womb Transplant Patient *BioNews* 2014; 740. Available at: http://www.bionews.org.uk/page_392763.asp [accessed 29 June 2015].

10
S. Dunlevy. Womb Transplants Coming to Australia in 2016 to Deliver the Chance of Pregnancy to Women without a Womb. *News*, 6 December 2014. Available at: http://www.news.com.au/lifestyle/health/womb-transplants-coming-to-australia-in-2016-to-deliver-the-chance-of-a-pregnancy-to-women-without-a-womb/story-fneuz9ev-1227146047542 [accessed 29 June 2015].

11

A. Nair et al. Uterus Transplant: Evidence and Ethics. Ann N Y Acad Sc 2008; 1127: 86. 10.1196/annals.1434.00318443334

12
C. Johnston. Womb Transplants: first 10 British women given go‐ahead. *The Guardian,* 30 September 2015. Available at: http://www.theguardian.com/lifeandstyle/2015/sep/29/10-women-receive-go-ahead-for-first-ever-womb-transplants-in-uk [accessed 16 October 2015].

13
Akar et al., *op. cit*. note 5, p. 1361.

14
M. Brannstrom et al. Livebirth after Uterus Transplantation. *Lancet* 2014; 385: 607‐616.; J. Laurance. Two Women Have Given Birth Using Wombs Transplanted from Their Mothers ‐ but Are the Risks Involved Too Great? *The Independent*, 3 December 2014. Available at: http://www.independent.co.uk/life-style/health-and-families/features/two-women-have-given-birth-using-wombs-transplanted-from-their-mothers-but-are-the-risks-involved-too-great-9901719.html [accessed 29 June 2015].

15
A number of commentators, for example, have asked: whether the procedure might cater to a faulty belief that certain kinds of parenthood (gestational and genetic) are more worth seeking than others (social); whether the prospective benefits to recipients really outweigh the harms it risks to the recipient, donor and potentially the resulting child; whether the uterus transplant really caters to the desire to ‘experience’ gestation and birth that it promises to, in light of the fact that current procedures at best result in gestation in an enervated uterus followed by caesarean section as opposed to vaginal delivery; whether the procedure is really a more desirable method of begetting a child than existing practices such as surrogacy; and finally, whether, in light of the high costs involved and its status as a life‐creating rather than life‐sustaining treatment, it should receive public funding in nations with fully or partially socialized medical systems.

Some, for example, have asked whether human research should have begun despite the fact that all attempts made so far at human UTx have preceded the life birth of an infant after allogeneic UTx in primates. Others have asked whether possible recipients can truly give consent to the procedure, at this current time, given the uncertainty of success and the strongly held desires it caters to.

16
Some, for example, have asked whether human research should have begun despite the fact that all attempts made so far at human UTx have preceded the life birth of an infant after allogeneic UTx in primates. Others have asked whether possible recipients can truly give consent to the procedure, at this current time, given the uncertainty of success and the strongly held desires it caters to.

17
Fageeh et al., *op. cit*. note 4, p. 247.

18

O. Ozkan et al. Preliminary Results of the First Human Uterus Transplantation from a Multiorgan Donor. Fertil Steril 2013; 99: 470. 2308426610.1016/j.fertnstert.2012.09.035

19
Brannstrom et al., *op. cit*. note 6, p. 1228.

20
S. Rivers. El ginecólogo César Díaz: El trasplante de útero beneficiaría a una de 4.000 mujeres en España. *20 Minutos España*, 7 October 2014. Available at: http://www.20minutos.es/noticia/2258498/0/ginecologo-espanol/primer-trasplante-utero/nacimiento-bebe/ [accessed 1 November 2015].

21
Kisu et al., *op. cit*. note 7.

22
Smith, *op. cit*. note 8.

23
Czub, *op. cit*. note 9.

24
Anon. Gent wil baarmoeders transplanteren. *De Standaard*, 11 December 2014. Available at: http://www.standaard.be/cnt/dmf20141210_01422982 [Accessed 1 November 2015].

25
Nair et al., *op. cit*. note 11, p. 86.

26

M. Brannstrom , C.A. Wranning & A. Altcheck . Experimental Uterus Transplantation. Hum Reprod Update 2010; 16: 340; Kisu et al., *op. cit*. note 7, p. 287. 10.1093/humupd/dmp04919897849

27

M. Brannstrom . Uterus Transplantation. Fertil Steril 2013; 99: 348. 2312295310.1016/j.fertnstert.2012.10.020

28
Kisu et al., *op. cit*. note 7, p. 288.

29
Brannstrom et al., *op. cit*. note 6, p. 1235.

30
Anon. Woman Gives Birth after Receiving Womb Transplant. *Irish Times*, 4 October 2014. Available at: http://www.irishtimes.com/news/health/woman-gives-birth-after-receiving-womb-transplant-1.1952400# [accessed 29 June 2015].

31
Brannstrom et al., *op. cit*. note 24, p. 340.

32
Brannstrom et al., *op. cit*. note 6, p. 1234.

33

G. Del Priore & D.K. Gudipudi . Promise of Uterine Transplant ‐ Myth or a Reality? Maturitas 2014; 7: 22. 10.1016/j.maturitas.2013.10.01224314618

34
Nair et al., *op. cit*. note 11, p. 86.

35
Ozkan et al., *op. cit*. note 18, p. 474.

36
Del Priore & Gudipudi, *op. cit*. note 31, p. 22.

37

M. Olausson et al. Ethics of Uterus Transplantation with Live Donors. Fertil Steril 2014; 102: 42. 10.1016/j.fertnstert.2014.03.04824784936

38
Brannstrom, *op. cit*. note 25, p. 348.

39
Kisu et al., *op. cit*. note 7, p. 288.

40
Brannstrom et al., *op. cit*. note 13, pp. 607‐616

41

FIGO Committee for the Ethical Aspects of Human Reproduction and Women's Health. FIGO Committee Report: Uterine Transplantation. Int J Gynaecol Obstet 2009; 106: 270. 19501356

42
Ibid.

43
NHS Blood And Transplant. 2014. Organ Donation and Transplantation Activity Report 2013/14. London: NHS: 37.

44
For examples of the few scholars who view that valid consent is not a necessary condition of morally permissible donation/research on living volunteers see: C. Fabre. 2006. *Whose Body Is It Anyway? Justice and the Integrity of the Person* Oxford: Clarendon Press; E. Rakowski. 1991. *Equal Justice*. Oxford: Clarendon Press.

45

J.S. Mill . 1977 . On Liberty In The Collected Works of John Stuart Mill vol. XVIII RobsonJ. M., ed. London: Routledge: 225.

46
Perhaps the most notable proponent of this view is John Harris who has suggested in numerous papers that on the basis of respect for individual autonomy the living should be permitted to donate even vital organs such as hearts. See, for example: J. Harris. 1992. *Wonderwoman and Superman ‐ the Ethics of Human Biotechnology*. Oxford: Oxford University Press: 113.

47
See, for example: W. Glannon & L.F. Ross. Response to ‘Intrafamilial Organ Donation Is Often an Altruistic Act’ by Aaron Spital (Cq Vol. 12, No. 1) and ‘Donor Benefit Is the Key to Justified Living Organ Donation’ by Aaron Spital (Cq Vol. 13, No. 1). *Camb Q Healthc Ethics* 2005; 14: 193.

48
I note that this tends to be the case in virtue of the fact that it may also rest on non‐aggregative considerations such as straightforward beneficence or some version of the rule of rescue.

49
See, for example: P. Reese et al. Creating a Medical, Ethical, and Legal Framework for Complex Living Kidney Donors. Clin J Am Soc Nephrol 2006; 1: 1150. 10.2215/CJN.0218060617699340

50
See, for example: E.J. Emanuel, D. Wendler & C. Grady.What Makes Clinical Research Ethical. *JAMA* 2000; 283: 2705 & R. Sauder & L.S. Parker. Autonomy's Limits: Living Donation and Health Related Harm. *Camb Q Healthc Ethics* 2001; 10: 405–406. 10.1017/s096318010100407814533406

51
See, for example: National Commission for the Protection of Human Subjects of Biomedical and Behavioural Research. 1978. *The Belmont Report: Ethical Principles and Guidelines for the Protection of Human Subjects of Research* Washington DC: Department of Health Education and Welfare: United States Government: 17 & Emanuel et al., *op. cit*. note 48, p. 2705. 25951677

52

A. Spital . Donor Benefit Is the Key to Justified Living Organ Donation. Camb Q Healthc Ethics 2004; 13: 108. 10.1017/s096318010413117415045922

53

C. Elliott . Doing Harm: Living Organ Donors, Clinical Research and the Tenth Man. J. Med Ethics 1995; 21: 91–96. 760894710.1136/jme.21.2.91PMC1376630

54
Sauder & Parker, *op. cit*. note 48, pp. 404–405.

55
Elliott, *op. cit*. note 51, p. 92.

56
Indeed, many scholars concerned with research ethics would view that the stronger requirement that participants must benefit from their participation may constitute undue inducement. Whether or not this is justified however, is up for debate and there is considerable disagreement among scholars concerning appropriate levels of compensation for research participants. See: M. Wilkinson & A. Moore. Inducement in Research. *Bioethics* 1997; 11: 373–389.

57
Emanuel et al., *op. cit*. note 48, p. 2705.

58
Should the reader strongly hold, like Harris, that this is not the case, it is suggested that a skip to the next section, which concerns issues of donor consent and regret, may be in order.

59
Spital, *op. cit*. note 50, p. 107.

60
See for example: L. Lechner , C. Bolman , & A. Van Dalen . Definite Involuntary Childlessness: Associations between Coping, Social Support and Psychological Distress. Hum Reprod 2007; 22: 288–294. 1692072210.1093/humrep/del327

61
Olausson et al., *op. cit*. note 35, p. 42.

62
Whilst some might question this suggestion during telephone calls with Mats Brannstrom (of the Swedish team) and Richard Smith (of the UK team) both informed the author that they have received numerous offers from women who would be willing to become altruistic living uterus donors for women with UFI.

63
It should be noted here that absent from this section is consideration of the question of whether the status of UTx as a quality of life as opposed to a vital organ transplant may cast doubt on the appropriateness of the use of living as opposed to deceased donors given that its purpose is not primarily to prolong lives, nor to treat a pathology that *necessarily* impacts on the quality of life of an organism as in most kidney transplants, but to treat a physical disorder whose impact on quality of life is contingent on the psychological attributes and desires of those who experience it. This critique of the use of living donors in UTx has not been fully unpacked within the literature although it was hinted at by Del Priore and Gudipudi when, in a paper regarding the deceased donor concept for UTx, they noted: ‘in general, the transplant community would never consider a living donor for anything less essential than a kidney or part of a liver.’ Whether or not this difference may cast doubt on the appropriateness of the use of living donors in UTx depends on whether a satisfactory argument can be provided in favour of the claim that the interests served and the benefits provided to the uterus recipient are *of less gravity* than those served and provided by vital organ transplantation. That such an argument can be provided however, is unlikely. That living donors are generally not utilized in QOL transplantation contexts likely has less to do with their status as QOL and more to do with the fact that one would be unlikely to find a willing living donor or a favourable harm‐benefit ratio in most other QOL contexts such as hand, face, cornea or larynx donation. This is not so with the possession of a uterus as for those who have already completed their families or who do not desire to gestate, the uterus is expendable. Indeed, the use of living donors in QOL transplantation contexts has already been sanctioned in the case of the ovary transplant (a similarly expendable organ) and so too is risk of harm commonly imposed on living volunteers in trials of medications and interventions that aim to provide purely psychological benefits to clinical populations. Consequently, it is unclear why the relatively novel status of UTx among transplantation procedures for which living donors are currently used should provide moral reason to prefer the use of deceased donors. See: Del Priore & Gudipudi, *op. cit*. note 31, p. 22.

64
Kisu et al., *op. cit*. note 7, p. 288.

65
Lefkowitz et al., *op. cit*. note 2, p. 433.

66

A. L. Caplan . Moving the Womb. Hastings Cent Rep 2007; 37: 19. 10.1353/hcr.2007.003617649898

67

R. Catsanos , W. Rogers & M. Lotz . The Ethics of Uterus Transplantation. Bioethics 2013; 27: 71. 10.1111/j.1467-8519.2011.01897.x21726265

68
Ibid.

69

O. O'Neill . Some Limits of Informed Consent. J. Med Ethics 2002; 29: 4–7. 1256918510.1136/jme.29.1.4PMC1733683

70
The content of the requirements that follow have been based on UK and international guidance regarding informed consent in both research and transplantation contexts as well as the criteria put forward in the Montreal Criteria For The Ethical Feasibility of Uterine Transplantation and the research design for the Swedish UTx Trial explored in various journal articles and at the COGI workshop on Uterine Transplantation. See, for example: Lefkowitz et al., *op. cit* note 2, pp. 439–447; Lefkowitz et al. Ethical Considerations in the Era of the Uterine Transplant: An Update of the Montreal Criteria for the Ethical Feasibility of Uterine Transplantation. *Fertil Steril* 2013; 100: 924–926; L. Johannesson. Inclusion/Exclusion Criteria of UTx Donors and Preoperative Investigations. at *The 19th World Congress on Controversies in Obstetrics, Gynaecology and Infertility 2014* Available at: https://www.youtube.com/watch?v=swXPnu-jtFo [accessed 29 June 2015]; National Commission for the Protection of Human Subjects of Biomedical and Behavioural Research, *op. cit* note 49, pp. 11–14; World Medical Association. Declaration of Helsinki. *Bull World Health Organ* 2001; 79: 373–374; Human Tissue Authority. 2015. *Guidance to Transplant Teams and Independent Assessors*. London: Department of Health.

71
For an exploration of the importance of institutional stability for surgical research and innovation see F. D. Moore . Ethical Problems Special to Surgery. Arch Surg 2000; 135: 14–16. 1063634010.1001/archsurg.135.1.14

72

Z. Mumtaz & A. Levay . Ethics Criteria for Uterine Transplants: Relevance for Low‐Income, Pronatalistic Societies?, J Clin Res Bioeth 2012; S1: 3.

73
Caplan, *op. cit*. note 63, p. 19.

74
NHS Blood and Transplant, *op. cit*. note 41, p. 120.

75
Epicurus. *Letter to Menoeceus*: 24‐25.

